# Exploring the role of serotonin as a plant stress modulator

**DOI:** 10.1080/15592324.2026.2627686

**Published:** 2026-02-09

**Authors:** Hamzeh Amiri

**Affiliations:** a Department of Biology, Faculty of Sciences, Lorestan University, Khorram Abad, Iran

**Keywords:** 5-hydroxytryptamine, melatonin, phytoserotonin, plant stress, oxidative stress, stress signaling

## Abstract

Serotonin (5-hydroxytryptamine), an indoleamine with a dual evolutionary legacy in animals and plants, has transcended its initial classification as a secondary metabolite to emerge as a central regulator of plant stress adaptation. This review moves beyond cataloging stress-associated effects to propose a unified framework for serotonin as a dynamic signaling and metabolic hub. I synthesize evidence that serotonin's role is defined not merely by its antioxidant capacity, but by its sophisticated integration into the core stress-signaling circuitry of plants. The key to this function is its inducible biosynthesis via the tryptophan decarboxylase (TDC) and tryptamine 5-hydroxylase (T5H) pathway, which is activated by diverse stressors through reactive oxygen species (ROS), phytohormone, and calcium-dependent signals. I critically analyze its multifaceted mechanisms: (1) direct and indirect ROS scavenging; (2) precise modulation of phytohormone networks (auxin, abscisic acid, jasmonic acid, salicylic acid), where it acts less as a hormone and more as a hormone signal modulator, notably fine-tuning root architecture and stomatal aperture; (3) regulation of ion transporter activity (e.g., SOS1, HMAs) for ionic homeostasis; and (4) epigenetic and transcriptional reprogramming of stress-responsive genes. A dedicated section clarifies the synergistic yet distinct partnership with melatonin, distinguishing serotonin's rapid, localized actions from melatonin's longer-term, systemic roles. I further explore serotonin's emerging functions in biotic stress as an antimicrobial compound and defense pathway potentiator. This integrative synthesis aims to reframe serotonin from a protective molecule to a master regulator at the nexus of plant stress perception and adaptive response.

## 1. Introduction

In the face of escalating global climate change, plants are increasingly subjected to a multitude of abiotic stressors, including drought, salinity, extreme temperatures, and heavy metal toxicity. These adverse conditions trigger complex physiological, biochemical, and molecular responses that determine plant survival, growth, and ultimately, agricultural productivity.^1^ Historically, research on plant stress adaptation has focused on classical phytohormones such as abscisic acid (ABA), jasmonic acid (JA), and salicylic acid (SA). However, the emerging role of bioactive small molecules, which are traditionally associated with animal systems, has opened a novel paradigm in plant stress biology. Among these, serotonin (5-hydroxytryptamine, 5-HT), a potent indoleamine, has garnered significant attention as a crucial regulatory metabolite with profound implications for plant resilience. It is imperative to distinguish serotonin from its metabolic derivative, melatonin; while they are biosynthetically linked and share some functional overlap, emerging evidence underscores unique, nonredundant roles for serotonin, particularly in rapid stress signaling and specific hormonal crosstalk.^2^^,^^3^

Serotonin is a phylogenetically ancient molecule, biosynthesized in plants from the amino acid tryptophan via the intermediates tryptamine and 5-hydroxytryptophan.^4^ Once considered a mere secondary metabolite, it is now recognized as a multifaceted signaling molecule with a ubiquitous presence across the plant kingdom. Its concentrations are highly dynamic, fluctuating markedly in response to various environmental cues. For instance, studies primarily employing exogenous application (typically in the 50–200 µM range) in species such as rice (*Oryza sativa*) and cucumber (*Cucumis sativus*) have documented rapid accumulation of serotonin or enhanced tolerance following exposure to drought, high salinity, and UV radiation, suggesting a role in early stress acclimation.^5^^,^^6^ Furthermore, endogenous biosynthesis is upregulated under stress, as shown by increased expression of tryptophan decarboxylase (TDC) and tryptamine 5-hydroxylase (T5H) genes in Arabidopsis and rice under pathogen attack or ionic stress.^1^^,^^7^

The proposed mechanistic roles of serotonin in abiotic stress mitigation are diverse, though the evidence base varies in depth. A well-documented function, supported by both in vitro assays and in planta studies, is its capacity to act as a direct antioxidant, scavenging reactive oxygen species (ROS) such as the hydroxyl radical.^2^ In addition, serotonin appears to intricately modulate the synthesis and signaling pathways of key phytohormones. Compelling evidence, primarily from Arabidopsis, indicates that it interferes with polar auxin transport by inhibiting PIN-FORMED (PIN) efflux carriers, strategically remodeling root architecture under stress.^3^ The interaction of ABA with ABA in promoting stomatal closure has been observed in species such as tomato and saffron, often using exogenous treatments.^8^^,^^9^ Claims regarding the modulation of ethylene or the reinforcement of physical barriers such as lignin, while promising, are currently supported by a more limited set of studies and require further validation across diverse species and physiological contexts.^10^

This manuscript aims to provide a comprehensive and critical synthesis of serotonin's role as a pivotal modulator of plant stress responses, with a primary focus on abiotic challenges while acknowledging its interconnected functions in biotic interactions. Through this structured and analytical approach, this review seeks to elevate the understanding of serotonin from a simple protective metabolite to that of a master regulatory hub in the plant stress-signaling network.

## 2. Biosynthesis and metabolism of serotonin in plants

**Figure 1.**
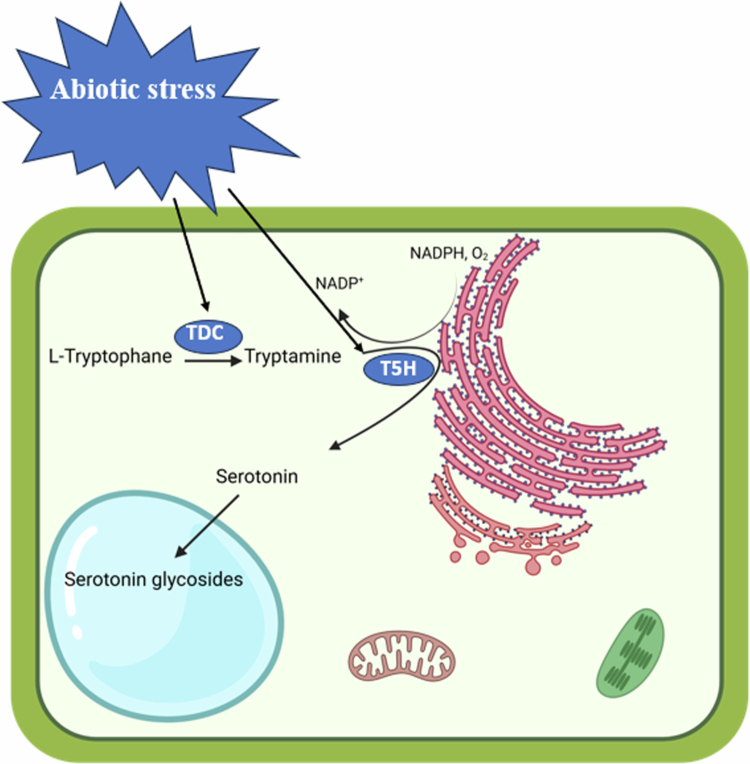
Abiotic stresses increase the endogenous serotonin content in plants by enhancing the activity of tryptophan decarboxylase (TDC) enzymes in the cytosol and tryptamine-5-hydroxylase (T5H) in the endoplasmic reticulum, leading to plant stress resistance responses.

Serotonin biosynthesis in plants occurs through a distinct tryptophan-dependent pathway that differs from the route observed in animals. The pathway initiates with the amino acid tryptophan, which undergoes decarboxylation by tryptophan decarboxylase (TDC) to form tryptamine.^11^^,^^12^ This cytosolic enzyme, requiring pyridoxal-5′-phosphate as a cofactor, has been characterized in various plant species, including rice (*Oryza sativa*) and *Catharanthus roseus*, showing strict substrate specificity for L-tryptophan.^5^^,^^13^ The subsequent hydroxylation of tryptamine at the 5-position of the indole ring is catalyzed by tryptamine 5-hydroxylase (T5H), a cytochrome P450 monooxygenase (CYP71A subfamily) that localizes to the endoplasmic reticulum.^14^^,^^15^ This membrane-bound enzyme requires molecular oxygen, NADPH, and cytochrome P450 reductase for activity, converting tryptamine to serotonin^16^^,^^17^ (Figure 1). The plant serotonin biosynthetic pathway contrasts with the mammalian system where tryptophan hydroxylation precedes decarboxylation, reflecting evolutionary divergence in indoleamine metabolism between kingdoms.^18^^,^^19^

Environmental stresses significantly influence serotonin biosynthesis through transcriptional regulation of TDC and T5H genes. Abiotic stressors such as drought, salinity, and extreme temperatures upregulate these biosynthetic enzymes, leading to increased serotonin accumulation.^7^^,^^13^ Similarly, biotic challenges including pathogen infection and herbivory induce serotonin production as part of the plant defense response.^14^ These stress-induced changes occur through complex signaling networks involving jasmonic acid, salicylic acid, and reactive oxygen species (ROS), with serotonin itself acting as a regulatory molecule that can modulate its own biosynthesis through feedback inhibition.^20^ Light quality and photoperiod also influence serotonin levels, with blue light particularly effective in stimulating T5H activity, suggesting integration of serotonin metabolism with photomorphogenic responses.^12^^,^^21^

Following its synthesis, serotonin undergoes various metabolic conversions that determine its physiological roles. The most characterized pathway involves its transformation to melatonin through sequential *N*-acetylation by serotonin *N*-acetyltransferase (SNAT) and O-methylation by acetylserotonin O-methyltransferase (ASMT).^22^ This metabolic link between serotonin and melatonin represents a critical branch point in indoleamine metabolism with significant implications for stress responses.^23^ Alternative metabolic fates include glycosylation by UDP-glycosyltransferases to form serotonin glucosides that accumulate in vacuoles, oxidative dimerization to form serotonin oligomers with enhanced antioxidant capacity, and conjugation with phenolic compounds to generate novel secondary metabolites (Figure 1). The relative flux through these competing pathways varies by tissue type and developmental stage, with chloroplast-containing tissues showing preferential conversion to melatonin while roots accumulate higher levels of serotonin conjugates.^14^^,^^16^

Subcellular compartmentalization plays a crucial role in serotonin metabolism, with biosynthesis occurring primarily in the cytoplasm while various metabolites localize to different organelles. Serotonin shows dynamic distribution patterns, accumulating in vacuoles as conjugated forms and moving through the vascular system to sink tissues.^13^^,^^24^ The hydrophobic nature of serotonin enables membrane diffusion, but specific transporters likely facilitate its movement between cellular compartments, particularly under stress conditions when rapid redistribution may be required.^21^ The pH-dependent ionization of serotonin influences its mobility in the apoplast and symplast, affecting its participation in both local and systemic signaling networks.^3^^,^^25^

The evolutionary conservation of serotonin biosynthesis across plant lineages suggests fundamental physiological importance, while lineage-specific expansions of TDC and T5H gene families indicate adaptive diversification.^13^^,^^15^ Comparative genomic analyzes reveal that all major plant groups maintain serotonin biosynthetic capacity, but with distinct regulatory features that may reflect ecological specialization.^14^^,^^18^ This evolutionary perspective informs current biotechnological applications where manipulation of serotonin biosynthesis through transgenic approaches has yielded crops with enhanced stress tolerance. Overexpression of TDC and T5H in model plants has demonstrated improved resistance to drought, salinity, and pathogen attack, validating serotonin's central role in stress adaptation.^19^^,^^26^

## 3. Serotonin as an integrative signal in abiotic stress: a mechanistic synthesis

**Figure 2.**
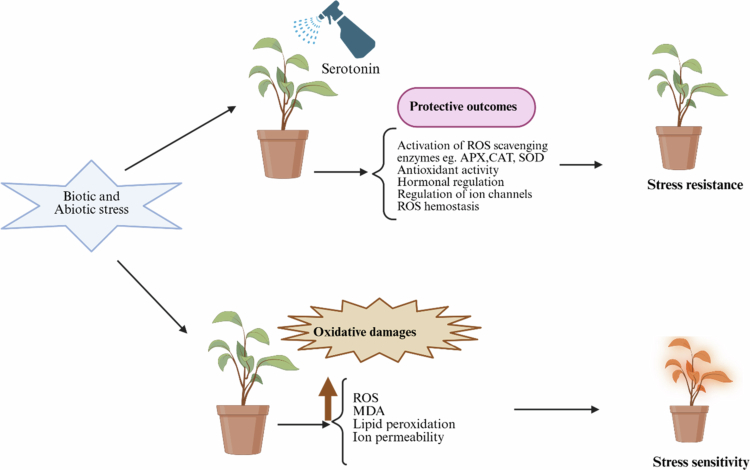
The effect of biotic and abiotic stresses on plants. The exogenous application of serotonin primarily induces stress resistance in plants through ROS homeostasis.

Serotonin serves a far more complex function in plant stress adaptation than merely neutralizing oxidative molecules, acting instead as a central coordinator that synchronizes a comprehensive defense strategy involving redox regulation, hormonal communication, genetic control, and ionic equilibrium.^27^ The integration of TDC and T5H begins with a dual-tiered antioxidant strategy, functioning as a direct chemical scavenger of reactive oxygen species (ROS) while simultaneously priming and upregulating the entire enzymatic antioxidant network, including superoxide dismutase, catalase, and the ascorbate‒glutathione cycle.^28^^,^^29^ Concurrently, serotonin acts as a sophisticated hormone signal modulator, fine-tuning critical phytohormone crosstalk. It recalibrates auxin transport and signaling to strategically remodel root architecture under stress, synergizes with abscisic acid (ABA) to promote stomatal closure while preventing excessive growth arrest, and balances jasmonic acid (JA) and salicylic acid (SA) pathways to prioritize context-appropriate defense outputs.^3^^,^^9^^,^^26^ At the genomic level, serotonin influences transcriptional and epigenetic reprogramming, modulating the expression of stress-responsive genes by regulating key transcription factor families—such as CBF/DREBs for cold acclimation and HSFs for heat tolerance—and inducing epigenetic modifications that establish a primed state for enhanced future resilience.^30^^-^^32^ Crucially, it directly regulates ion transport machinery, enhancing sodium exclusion via the SOS pathway under salinity and promoting vacuolar sequestration of toxic metals through the upregulation of Heavy Metal ATPases (HMAs) and phytochelatin synthesis.^1^^,^^33^ The true sophistication of serotonin lies in its context-dependent application of this integrated toolkit; it strategically emphasizes specific mechanisms—such as water economy via ABA-auxin crosstalk during drought, ionic homeostasis via transporter regulation during salinity, or direct chelation combined with genetic detoxification during heavy metal stress—based on the precise environmental cue.^8^^,^^17^ This ability to interpret stress signals and coordinately activate the most appropriate combination of biochemical, hormonal, genetic, and ionic adjustments elevates serotonin from a mere protective metabolite to a central processing hub in the plant stress-signaling network, enabling coherent, whole-organism adaptations that optimize the critical trade-off between immediate survival and sustainable growth under adverse conditions (Figure 2).

### 3.1. Redox homeostasis: a two-tiered system of serotonin action

The management of cellular redox equilibrium under stress involves a complex, two-tiered defensive approach that goes beyond basic antioxidant functions. This system merges rapid, direct chemical neutralization with the prolonged, system-wide activation of the plant's innate enzymatic protective systems, positioning serotonin as a principal controller of oxidative balance. The first tier leverages serotonin's inherent biochemical properties as a potent direct scavenger of reactive oxygen species (ROS). Its molecular architecture, featuring an electron-rich indole ring and a hydroxyl group, confers a high capacity for electron donation, enabling efficient neutralization of highly damaging radicals such as the hydroxyl radical (•OH), with a rate constant comparable to classical antioxidants like melatonin.^34^ This direct action extends to quenching superoxide anions (O₂•⁻), hydrogen peroxide (H₂O₂), and lipid peroxyl radicals (ROO•), providing a crucial first line of rapid, nonenzymatic defense during the initial oxidative burst that accompanies stress perception.^2^ For instance, in cucumber seedlings subjected to chilling stress, exogenous serotonin application led to measurable and rapid decline in H₂O₂ and •OH accumulation, directly preserving membrane integrity within hours of treatment.^6^

The second, and arguably more impactful, tier involves serotonin's function as a signaling molecule that primes and transcriptionally reprograms the plant's entire antioxidant network. This represents a shift from acting as a sacrificial antioxidant to serving as a regulator of long-term redox resilience. Serotonin orchestrates a broad upregulation of genes encoding core enzymatic antioxidants. Transcriptomic analyzes across species consistently show that serotonin treatment enhances the expression of genes for superoxide dismutase (SOD), which dismutates O₂•⁻ to H₂O₂; catalase (CAT) and various peroxidases like ascorbate peroxidase (APX) and glutathione peroxidase (GPX), which decompose H₂O₂; and glutathione reductase (GR), which regenerates reduced glutathione (GSH).^26^^,^^28^ Crucially, serotonin amplifies the ascorbate-glutathione (AsA-GSH) cycle, the central hub for H₂O₂ detoxification in chloroplasts and the cytosol, by boosting the activity and/or expression of its component enzymes—APX, monodehydroascorbate reductase (MDHAR), dehydroascorbate reductase (DHAR), and GR.^29^^,^^35^ Beyond enzymes, serotonin elevates the cellular pools of key antioxidant metabolites, increasing the reduced-to-oxidized ratios of both glutathione (GSH/GSSG) and ascorbate (AsA/DHA), thereby maintaining a highly reducing cellular environment essential for protecting proteins and membranes.^31^ Furthermore, serotonin fine-tunes ROS signaling itself, potentially modulating the activity of NADPH oxidases (RBOHs) to ensure that stress-induced ROS bursts are sufficient to activate defense pathways like stomatal closure without escalating to toxic levels.^20^

The synergistic effect of these two tiers is a marked and consistent reduction in oxidative damage biomarkers across diverse stresses. Studies in drought-stressed wheat, salt-stressed rice, and cadmium-exposed plants uniformly report that serotonin application not only directly scavenges ROS but also significantly increases the activities of SOD, CAT, and APX, leading to a substantial decrease in malondialdehyde (MDA) content and electrolyte leakage, definitive indicators of reduced lipid peroxidation and membrane damage.^1^^,^^8^^,^^33^ This integrated action reframes serotonin from a mere antioxidant to a redox homeostasis regulator. Its direct chemical scavenging provides immediate buffering capacity, while its signaling role induces a state of systemic acquired acclimation. This priming effect means serotonin-treated plants are not only protected at the moment of application but are biochemically “tuned” to respond more robustly and efficiently to subsequent stress events—a phenomenon increasingly linked to the epigenetic modifications that serotonin can influence, such as altering DNA methylation patterns at the promoters of stress-responsive genes.^30^ Thus, serotonin's management of redox homeostasis is a comprehensive, adaptive strategy that combines rapid-response capability with the long-term, programmable capacity of a signaling molecule that dynamically reprograms the plant's own defense network, a functionality central to its role as a cornerstone of plant stress tolerance (Figure 2).

### 3.2. Phytohormone network modulation: the integrative hub of serotonin signaling

Serotonin’s most sophisticated and defining role in plant stress adaptation lies not in its independent action, but in its profound ability to modulate, integrate, and fine-tune the established phytohormone signaling networks. It functions as a master orchestrator or “rheostat” within the hormonal symphony, calibrating the balance between growth, defense, and stress adaptation. This modulation is not generic but involves precise, molecular-level interactions with key hormonal pathways. The most well-characterized interaction is with auxin, where serotonin functions as a context-dependent modulator rather than a mere mimic or inhibitor. Serotonin directly interferes with polar auxin transport by inhibiting the activity of PIN-FORMED (PIN) auxin efflux carriers, particularly PIN1 and PIN2, which are critical for establishing auxin gradients in the root tip.^3^ This disruption diminishes the auxin maximum at the root apex, leading to the inhibition of primary root elongation. Concurrently, serotonin influences auxin signaling and homeostasis, altering the expression of auxin-responsive genes and potentially competing with indole-3-acetic acid (IAA) for binding sites or interfering with TIR1/AFB auxin receptor complex formation.^18^ Paradoxically, while inhibiting primary growth, serotonin stimulates the production of lateral and adventitious roots by promoting auxin accumulation in specific pericycle cells, possibly through localized transport inhibition or interaction with cytokinin pathways.^30^ This precise modulation allows for strategic remodeling of root system architecture under stress, enabling adaptations like a shallower, branched system for drought or reduced penetration into metal-contaminated soil layers.

The relationship between serotonin and abscisic acid (ABA) is characterized by context-dependent synergy and calibration. Under drought stress, serotonin enhances ABA-induced stomatal closure by potentiating signaling components downstream of PYR/PYL/RCAR receptors, increasing cytosolic Ca²⁺ oscillations and reactive oxygen species in guard cells to amplify the signal for ion efflux.^8^^,^^9^ Serotonin also regulates ABA metabolism, upregulating biosynthetic genes like *NCED* under drought while potentially modulating catabolic genes to prevent excessive growth arrest under prolonged stress.^1^ In roots under salt or osmotic stress, serotonin's growth-promoting effects can partially counteract severe ABA-induced growth inhibition, performing a homeostatic balancing act to ensure survival processes do not completely halt exploratory growth. In biotic stress responses, serotonin acts as a signaling integrator that helps prioritize the appropriate defense pathway. It strongly potentiates the salicylic acid (SA) pathway effective against biotrophic pathogens by upregulating the central regulator NPR1 and pathogenesis-related genes like *PR1.*^7^ Its interaction with jasmonic acid (JA) is more complex and often antagonistic; in rice infected with *Magnaporthe oryzae*, serotonin accumulation suppresses JA-responsive gene expression, strategically allocating resources to the more effective SA pathway.^26^ Serotonin likely targets known integration nodes between JA and SA, such as WRKY transcription factors or MAP kinase cascades, possibly stabilizing NPR1 in its active form or influencing JAZ protein degradation in a pathogen-specific manner.^31^

Emerging evidence points to broader hormonal integration with ethylene and cytokinins. Serotonin influences ethylene biosynthesis and signaling, with some of its root growth regulation effects mediated through changes in ethylene production, creating a tripartite interaction (serotonin-auxin-ethylene) that finely controls root morphology.^20^ Under stress, serotonin may help maintain cytokinin signaling, which promotes cell division and delays senescence, linking to its documented role in delaying leaf senescence in rice through modulation of both cytokinin and ABA levels.^5^ This detailed analysis supports reframing serotonin not as a new phytohormone with a linear pathway, but as a hormone signal modulator (HSM). Its functions are context-specific, with effects (synergistic or antagonistic) depending on the hormone, tissue, and stress type. It operates upstream and parallel to classical hormones, modifying their perception, transport, or early signaling events. Most importantly, it is integrative, allowing the plant to compute multiple stress signals and output a coordinated hormonal response that optimizes the trade-off between growth, abiotic tolerance, and biotic defense. By precisely adjusting the gains on auxin, ABA, JA, and SA signaling pathways, serotonin enables a plastic, efficient, and highly regulated response to an unpredictable environment, elevating it from a mere protective metabolite to a central hub in the plant stress-signaling interactome.

### 3.3. Transcriptional and epigenetic regulation by serotonin: programming stress resilience

The impact of serotonin reaches past direct biochemical effects to fundamentally influence the plant's genetic and epigenetic framework, serving as a regulator of transcription and an initiator of epigenetic changes that reconfigure gene expression patterns to confer both short-term stress resistance and enduring adaptive potential. This represents one of the most sophisticated frontiers in understanding serotonin's role as a master stress regulator. At the transcriptional level, serotonin functions as a signaling molecule that directly or indirectly influences the activity of key transcription factor families and the expression of broad suites of stress-responsive genes. Under cold and osmotic stress, serotonin application upregulates the expression of C-repeat Binding Factors (CBFs/DREB1s) in Arabidopsis and rice, which activate a cascade of Cold-Regulated (COR) genes involved in osmoprotection and membrane stabilization.^32^ During heat stress, serotonin promotes the accumulation of Heat Shock Proteins (HSPs) by activating Heat Shock Transcription Factors, particularly HSFA1s and HSFA2s, with transcriptomic data from tomato indicating that serotonin pretreatment enhances the expression of HSFA2 and its downstream targets HSP70 and HSP90, crucial for maintaining proteostasis.^31^ In biotic stress contexts, serotonin influences the expression of numerous WRKY transcription factors, which are central hubs in defense signaling; for example, in rice under pathogen attack, serotonin accumulation correlates with the upregulation of WRKY45, a key positive regulator of salicylic acid-mediated defense.^7^ Global transcriptome analyzes consistently reveal serotonin-mediated upregulation of functional gene categories including osmoprotectant biosynthesis (e.g., proline via P5CS, glycine betaine via BADH), Late Embryogenesis Abundant (LEA) proteins and dehydrins that protect cellular structures from dehydration damage, detoxification enzymes like glutathione S-transferases (GSTs), and pathogenesis-related (PR) proteins such as PR1 and PR2 in defense scenarios.^29^^,^^30^

Perhaps the most groundbreaking aspect of serotonin's action is its emerging role in epigenetic regulation, potentially enabling transgenerational stress memory through mechanisms that alter gene expression without changing the DNA sequence. Serotonin can influence DNA methylation patterns, a key epigenetic mark typically associated with gene silencing. In Arabidopsis and wheat under heavy metal stress, exogenous serotonin treatment was shown to alter genome-wide DNA methylation patterns, inducing hypomethylation at the promoter regions of key stress-responsive genes like SOD, P5CS, and HMA3 to activate their expression while potentially promoting hypermethylation at promoters of genes involved in negative regulators of stress responses.^30^^,^^31^ Serotonin is also implicated in modifying histone tails to change chromatin conformation, potentially influencing the activity of Histone Acetyltransferases (HATs) to increase histone H3 and H4 acetylation at stress gene loci, thereby loosening chromatin and facilitating transcription factor binding for rapid gene activation under heat stress.^2^ Evidence suggests serotonin can affect histone methylation marks, promoting H3K4me3 activation marks at defense gene promoters while modulating repressive H3K27me3 marks at developmental genes that need temporary silencing under stress.^17^ Furthermore, serotonin influences the biogenesis or activity of microRNAs (miRNAs) and small interfering RNAs (siRNAs) that guide post-transcriptional gene silencing and chromatin modifications; under cadmium stress, serotonin treatment in rice altered expression profiles of miRNAs targeting transcripts for nutrient transporters and oxidative stress enzymes, fine-tuning the post-transcriptional landscape of the stress response.^33^

The combined transcriptional and epigenetic actions of serotonin contribute significantly to the phenomenon of priming or acquired stress resistance, where a plant previously exposed to serotonin displays a faster, stronger, and more energy-efficient response to subsequent severe stress. The molecular basis of this priming involves the epigenetic marks and elevated baseline levels of certain transcription factors established by serotonin, creating a “primed state” where genes can be activated more rapidly upon secondary stress, leading to quicker accumulation of protectants like HSPs or antioxidants.^29^ While direct evidence for serotonin-induced transgenerational epigenetic inheritance in plants remains limited, the precedent exists for stress-induced epigenetic changes, making it a compelling hypothesis that serotonin-mediated modifications in reproductive tissues could confer enhanced stress tolerance to offspring. This layer of control, acting over hours to potential generations, underscores serotonin's evolutionarily sophisticated role as a programmer of cellular stress memory. By recruiting transcription factors to specific gene promoters and establishing permissive or repressive epigenetic landscapes, serotonin does not just help the plant survive the current stress—it fundamentally rewires its genetic response machinery for greater resilience against future challenges, an capability that far surpasses the functions of a simple antioxidant molecule and positions it as a central regulator of plant adaptation.

### 3.4. Ion homeostasis and transport regulation: the orchestration of cellular equilibrium by serotonin

The regulation of ion homeostasis is a core, though frequently overlooked, component of serotonin's function as a principal stress regulator. This capability functions not via a single pathway but through a coordinated suite of actions that work in concert to preserve ionic and osmotic equilibrium during environmental stress. The molecule's influence spans from the direct modulation of specific transporter proteins and channels to the orchestration of broader signaling pathways that govern cellular ion fluxes. Under salinity stress, serotonin exerts a profound influence on the critical balance between sodium and potassium ions. It achieves this by positively regulating the salt overly sensitive (SOS) pathway, a cornerstone of plant salt tolerance. Exogenous serotonin application has been demonstrated to upregulate the expression of SOS1, which encodes the plasma membrane Na⁺/H⁺ antiporter responsible for extruding sodium from the cytosol.^1^^,^^9^ This transcriptional enhancement provides more molecular machinery for sodium exclusion. Concurrently, serotonin likely potentiates the activity of the SOS2/SOS3 kinase complex, the calcium-sensitive activator of SOS1, ensuring that the antiporter is fully functional in response to salt-induced calcium signals.^29^ To energize this efflux, serotonin stimulates the plasma membrane H⁺-ATPase (PMA), strengthening the proton motive force that drives SOS1.^36^ On the potassium front, serotonin acts to preserve cellular K⁺ pools, which are crucial for enzymatic activity and turgor maintenance. It mitigates salt-induced K⁺ leakage, potentially by modulating outward-rectifying K⁺ channels (GORK), and promotes K⁺ influx by upregulating high-affinity K⁺ transporters (e.g., HAK5) in root tissues, thereby actively countering the antagonistic effects of Na⁺ on K⁺ uptake.^17^^,^^37^

In the context of heavy metal stress, serotonin deploys a sophisticated, multilayered defense strategy centered on chelation, exclusion, and sequestration. Its indoleamine structure, featuring amine and hydroxyl functional groups, enables it to act as a direct chelator of toxic metal cations such as Cd²⁺, Pb²⁺, and Al³⁺. By forming stable complexes in the apoplast and rhizosphere, serotonin reduces the bioavailability and subsequent uptake of these metals, as evidenced in pea plants, where serotonin-Cd complexes in root cell walls decreased translocation to shoots.^33^^,^^38^ Complementing this chemical defense, serotonin influences the genetic machinery of metal transport. It can suppress the expression of broad-specificity influx transporters such as natural resistance-associated macrophage proteins (NRAMPs), which are primary conduits for cadmium and manganese entry.^30^ For metals that do enter the cytosol, serotonin promotes their safe compartmentalization. It upregulates genes encoding heavy metal ATPases (HMAs), such as tonoplast-localized HMA3, which actively pumps Cd and Pb into the vacuole, effectively detoxifying the cytosol.^31^ Furthermore, serotonin enhances the synthesis of phytochelatins (PCs) by upregulating phytochelatin synthase (PCS) genes. These metal-binding peptides form complexes with cytosolic metals, which are then trafficked into vacuoles via ABCC transporters, creating a synergistic intracellular detoxification pathway.^27^

Underpinning these targeted actions on specific transporters is serotonin's broader modulation of secondary messenger systems that govern ion transport. The molecule influences cytosolic calcium ([Ca²⁺]cyt) signatures by potentially affecting calcium channel (e.g., CNGCs) and pump (e.g., ACA) activities. Since many stress-activated transporters, including SOS1, are regulated by calcium-dependent phosphorylation, serotonin's influence on Ca²⁺ dynamics provides a foundational control layer.^20^ Similarly, by regulating H⁺-ATPase activity, serotonin directly impacts apoplastic and cytosolic pH, which in turn affects the charge state and activity of numerous ion channels and the binding capacity of cell walls for cations. The integrated outcome of this sophisticated regulatory network is the robust preservation of cellular integrity under ionic stress. Under salinity, this manifests as reduced cytosolic Na⁺, maintained K⁺ levels, and a healthy membrane potential. Under heavy metal stress, it results in minimized metal uptake and maximal vacuolar sequestration, protecting vital metabolic and photosynthetic processes. Conceptually, serotonin functions as a cellular gatekeeper and traffic director, dynamically closing harmful influx pathways, opening efficient efflux and sequestration routes, and maintaining essential nutrient flows. This precise, multitarget coordination of ion transport machinery is not a peripheral effect but a core mechanism that underscores serotonin's evolutionarily sophisticated role as a central regulator of plant adaptation to environmentally imposed ionic challenges (Figure 2).

## 4. Applying the framework: stress-specific nuances

While serotonin's core mechanisms provide a unified framework for stress adaptation, their application is not uniform. The plant dynamically prioritizes and combines these mechanisms in response to specific environmental cues. This section details how the integrative framework of serotonin action is tailored to counteract the unique physiological challenges posed by each major abiotic stressor.

### 4.1. Serotonin action under drought stress

The role of serotonin in optimizing water economy in plants is very important and can be exerted through the following mechanisms:

**Primary hormonal integration:** Crosstalk with abscisic acid (ABA) is paramount. Serotonin potentiates ABA signaling in guard cells, enhancing the sensitivity and speed of stomatal closure to minimize transpirational water loss.^8^^,^^9^ Concurrently, its well-characterized modulation of auxin signaling initiates a strategic remodeling of the root system architecture (RSA). By acting as a natural auxin inhibitor, serotonin suppresses primary root elongation while stimulating lateral and adventitious root proliferation.^3^ This fosters a shallower, more extensively branched root network optimized for exploring soil moisture in the upper horizons where water persists longer after light rainfall or irrigation.

**Redox and osmotic management:** The oxidative burst triggered by cellular dehydration is mitigated by serotonin's two-tiered antioxidant system, protecting membranes and proteins. Furthermore, serotonin upregulates the biosynthesis of compatible solutes, notably proline and soluble sugars, contributing to osmotic adjustment and the stabilization of cellular structures to maintain turgor under water deficit.^29^

**Transcriptional reprogramming:** Serotonin influences the expression of drought-responsive genes, including those encoding late embryogenesis-abundant (LEA) proteins and dehydrins, which protect cellular components from desiccation damage. It also modulates genes involved in cuticular wax biosynthesis, adding another layer of protection against nonstomatal water loss.^30^

In essence, under drought, serotonin functions as a central coordinator of the classic survival trade-off: minimizing water loss (via ABA/stomata) while maximizing water capture (via auxin/root architecture), all while maintaining cellular integrity through redox and osmotic control.

### 4.2. Serotonin action under salinity stress

Salinity stress presents a triple threat: ionic toxicity (Na⁺), osmotic stress, and secondary oxidative damage. Serotonin acts in the following ways to manage ion homeostasis for plant tolerance to salt stress.

**Ion transport regulation:** serotonin's most critical role under salinity is the regulation of the salt overly sensitive (SOS) pathway. It upregulates the expression and activity of SOS1, the plasma membrane Na⁺/H⁺ antiporter, to actively extrude sodium from the cytosol.^1^ This action is energized by serotonin's stimulation of the plasma membrane H⁺-ATPase (PMA), which strengthens the proton motive force.^36^ Concurrently, serotonin works to preserve cytosolic K⁺ by promoting high-affinity K⁺ uptake (e.g., via HAK transporters) and suppressing stress-induced K⁺ leakage, thereby maintaining the vital K⁺/Na⁺ ratio.^17^^,^^37^

**Hormonal and redox fine-tuning:** the interaction with ABA under salinity is more nuanced than that under drought. Serotonin may fine-tune ABA sensitivity to prevent excessive growth arrest while still utilizing ABA-mediated signals for partial stomatal regulation and the activation of compatible solute synthesis. The antioxidant defense is crucial but specifically targets the oxidative damage secondary to sodium influx and osmotic shock, with a pronounced protective effect on the photosynthetic apparatus.^28^

**Root system adaptation:** while root growth is often inhibited by salt, serotonin's modulation of auxin can help maintain root meristem activity and promote branching in less saline soil patches, allowing for compensatory nutrient and water uptake.^3^ Thus, under salinity, serotonin acts primarily as a cellular gatekeeper, prioritizing the exclusion of Na⁺, retention of K⁺, and mitigation of ion-driven oxidative stress to preserve metabolic function.

### 4.3. Serotonin action under heavy metal stress

Toxic heavy metals such as cadmium (Cd), lead (Pb), and arsenic (As) require a unique defensive portfolio where direct chemical intervention complements genetic and physiological adaptations.

**Direct chemical defense (Chelation):** A distinctive mechanism is serotonin's capacity for extracellular chelation. Its indoleamine structure allows it to form stable complexes with metal cations in the rhizosphere and root apoplast, directly reducing their bioavailability and uptake.^33^ This is a first line of chemical defense, as demonstrated in pea plants where serotonin-Cd complexes in root cell walls decreased translocation to shoots.^38^

**Genetic and transport-based detoxification:** For metals that enter the cytosol, serotonin activates the intracellular sequestration machinery. It upregulates genes for phytochelatin (PC) synthesis and tonoplast-localized heavy metal ATPases (e.g., HMA3) to pump toxic ions into the vacuole, effectively detoxifying the cytoplasm.^31^ It may also repress the expression of broad-specificity metal influx transporters, such as NRAMPs.^30^

**Integrated protective responses:** Serotonin's robust antioxidant system mitigates the severe oxidative burst induced by metals. The modulation of auxin signaling can promote a root architecture that minimizes growth into heavily contaminated soil zones. Emerging evidence also highlights a role in inducing epigenetic modifications (e.g., DNA hypomethylation at metal detoxification gene promoters), which may provide a lasting adaptive imprint in contaminated environments.^30^

### 4.4. Serotonin action under temperature stress (heat and cold)

Temperature extremes disrupt membrane fluidity, protein stability, and metabolic harmony. Serotonin's protective strategy emphasizes the stabilization of macromolecular structures and the regulation of specific acclimation pathways.

**Heat stress response:** beyond countering heat-induced oxidative damage, serotonin is critical for proteostasis. It induces the expression of molecular chaperones, particularly heat shock proteins (HSP70, HSP90), which prevent the aggregation of denatured proteins and facilitate refolding.^31^ Serotonin also contributes to membrane stabilization, helping to maintain lipid bilayer integrity and fluidity at elevated temperatures.

**Cold stress response:** serotonin engages the CBF/DREB transcriptional cascade, a master switch for cold acclimation. By upregulating CBF expression, it promotes the synthesis of cryoprotective compounds such as proline, soluble sugars, and specific LEA proteins.^32^ It also influences calcium signaling pathways associated with cold perception and modulates ABA signaling to coordinate the cold acclimation process.

**Common protective outcomes:** for both heat and cold, a vital outcome of serotonin action is the preservation of photosynthetic efficiency. It helps protect chlorophyll, maintain photosystem II (PSII) complex stability, and safeguard the electron transport chain, ensuring that energy production continues despite thermal challenge.^29^

Therefore, under temperature stress, serotonin acts as a guardian of cellular infrastructure, prioritizing protein and membrane stability, activating specialized genetic acclimation programs, and ensuring the continuity of photosynthesis (Table 1).

**Table 1.** Key physiological mechanisms of serotonin in plant stress tolerance.

Physiological characteristic	Effect of serotonin under Stress	Species/tissue studied	Specific endpoint measured	Direction of effect	Mechanism and outcome	Key references
Photosynthetic efficiency	Protects and preserves photosynthetic capacity.	Rice (*Oryza sativa*) leaves; Cucumber (*Cucumis sativus*) seedlings.	Chlorophyll a/b content, Fv/Fm ratio (PSII quantum yield), net photosynthetic rate (Pn).	Increase	Scavenges ROS generated by stress-induced photoinhibition, protecting photosystem II (PSII) and preventing chlorophyll degradation. Maintains higher pigment content and photochemical efficiency.	[6,29]
Water relations and osmotic balance	Improves water use efficiency (WUE) and maintains turgor.	Saffron (*Crocus sativus*) leaves; tomato (*Solanum lycopersicum*) seedlings.	Stomatal conductance (gs), transpiration rate, relative water content (RWC), proline content.	Decrease (gs); increase (RWC, proline)	Modulates stomatal aperture, reducing water loss during drought. Promotes the synthesis of osmoprotectants (e.g., proline) to maintain cellular osmotic potential.	[8,9]
Ion homeostasis	Mitigates ion toxicity and maintains nutrient balance.	Rice (*Oryza sativa*) roots/shoots; Tomato seedlings.	Tissue Na⁺ and K⁺ content, Na⁺/K⁺ ratio, expression of *SOS1* and *HAK* transporters.	Decrease (Na⁺/K⁺ ratio); Increase (K⁺ content, transporter expression)	Under salinity stress, upregulates the SOS pathway for Na⁺ exclusion and enhances high-affinity K⁺ uptake, maintaining a favorable K⁺/Na⁺ ratio crucial for enzymatic activity.	[1,17]
Membrane stability	Reduces membrane lipid peroxidation.	Wheat (*Triticum aestivum*) leaves; Arabidopsis thaliana.	Malondialdehyde (MDA) content, electrolyte leakage (EL).	Decrease	Potent antioxidant activity neutralizes free radicals that attack lipid membranes, reducing oxidative damage biomarkers and preserving membrane integrity.	[2,8]
Antioxidant enzyme activity	Modulates the plant's enzymatic antioxidant defense system.	Rapeseed (*Brassica napus*) leaves; Rice seedlings.	Activity of SOD, CAT, APX, GR; redox state of ascorbate (AsA/DHA) and glutathione (GSH/GSSG).	Increase	Upregulates the activity and/or expression of key enzymes (SOD, CAT, APX, GR) and enhances the ascorbate-glutathione cycle, boosting the cell's capacity to neutralize ROS.	[28,29,39]
Defense signaling and gene expression	Primes immune and defense responses.	Rice (*Oryza sativa*) leaves; Arabidopsis thaliana.	Expression of *PR1*, *NPR1*, *WRKY* TFs; lignin content; callose deposition.	Increase	Acts as a signaling molecule triggering defense gene expression and the phenylpropanoid pathway, enhancing resistance against biotic stressors.	[7,31]
Nutrient uptake and detoxification	Influences nutrient metabolism and heavy metal detoxification.	Rice seedlings; Pea (*Pisum sativum*) roots.	Cd/Pb uptake, phytochelatin (PC) content, expression of *HMA3* and *PCS*.	Decrease (metal uptake); Increase (PCs, gene expression)	Under heavy metal stress, can chelate metals extracellularly and upregulate genes for intracellular sequestration (e.g., HMAs, PCS), reducing metal toxicity.	[30,33,40]

## 5. Serotonin in biotic stress and the phytobiome: a multidimensional defense strategy

In the realm of biotic interactions, serotonin transitions from a modulator of abiotic stress to a proactive and versatile defense compound, orchestrating a multilayered strategy that encompasses direct antimicrobial action, immune system potentiation, and sophisticated communication within the phytobiome. Unlike its nuanced hormonal crosstalk under abiotic conditions, serotonin's role here often involves direct chemical confrontation. It exhibits intrinsic antimicrobial and antifeedant properties, leveraging its chemical structure to disrupt pathogen integrity. Research on rice blast disease demonstrates that serotonin accumulation inhibits the growth of *Magnaporthe oryzae* through mechanisms likely involving fungal membrane disruption and the induction of oxidative stress within the pathogen cells themselves.^7^^,^^41^ Similar antifungal efficacy has been documented against pathogens like *Botrytis cinerea* in tomato and *Fusarium graminearum* in wheat, where exogenous serotonin application can reduce disease severity by 40−60%, highlighting its potential as a natural alternative to synthetic fungicides.^28^ Against herbivores, serotonin acts as a neuroactive feeding deterrent; in maize, it can bind to insect neural receptors, disrupting normal feeding behavior and providing a direct chemical defense.^2^

Beyond direct toxicity, serotonin functions as a powerful immune potentiator, enhancing the plant's innate defensive capabilities. It acts as a priming agent for pattern-triggered immunity (PTI) by upregulating the expression of pattern recognition receptors (PRRs), such as FLS2 and EFR in Arabidopsis.^31^ This priming results in a stronger and faster defensive response upon pathogen encounter, characterized by amplified reactive oxygen species (ROS) bursts, enhanced callose deposition at cell walls, and rapid stomatal closure to physically block bacterial entry. At the molecular level, serotonin extensively modulates the phytohormonal circuitry of defense, exhibiting a synergistic relationship with salicylic acid (SA) to bolster defenses against biotrophic pathogens while often antagonizing jasmonic acid (JA) signaling in specific pathosystems to strategically prioritize the most effective defense pathway.^26^ In Arabidopsis, serotonin accumulation potentiates SA-mediated systemic acquired resistance (SAR) by enhancing the expression of the central regulator NPR1 and pathogenesis-related genes like *PR1.*^3^

Perhaps the most innovative dimension of serotonin's role in biotic stress is its emerging function as a modulator of the phytobiome. Serotonin is not confined to intracellular signaling; it is actively exuded into the rhizosphere, where it acts as a chemical signal that shapes microbial community composition. Studies on Arabidopsis indicate that root exudation of serotonin can alter the structure of the rhizosphere microbiome, selectively enriching for beneficial plant growth-promoting rhizobacteria (PGPR) that possess pathogen-suppressive traits or enhance overall plant health.^17^^,^^29^ This represents a novel form of indirect defense, where serotonin helps cultivate a protective microbial shield in the soil environment. Furthermore, serotonin contributes to physical barrier formation at infection sites, promoting processes like lignification and suberization of cell walls to hinder pathogen penetration, and can influence the emission of herbivore-induced plant volatiles (HIPVs) that attract natural enemies of pests, adding an ecological layer to its defensive repertoire.^41^^,^^42^

In conclusion, serotonin's involvement in biotic stress is multidimensional and ecologically integrated. It operates simultaneously as a direct toxin, an immune system primer, a hormonal network orchestrator, and a rhizosphere ecologist. This capacity to coordinate direct chemical defense with induced systemic resistance and microbiome management underscores serotonin's evolutionary significance as a central regulator of plant health in a complex biotic world. Its ability to function across these levels—from cellular chemistry to community ecology—makes it a uniquely promising target for developing sustainable, multifaceted crop protection strategies that enhance innate plant immunity while fostering beneficial below-ground alliances (Table 1).

## 6. Distinguishing the serotonin‒melatonin partnership: synergy through division of labor

The relationship between serotonin and melatonin in plant stress adaptation is often described as a partnership, but this term belies a sophisticated, sequential, and functionally specialized relationship characterized by a clear division of labor that creates synergistic outcomes. Fundamentally, this partnership is rooted in an obligatory metabolic link: serotonin serves as the direct biochemical precursor to melatonin, undergoing sequential *N*-acetylation by serotonin *N*-acetyltransferase (SNAT) and O-methylation by acetylserotonin O-methyltransferase (ASMT) to yield melatonin.^22^ This precursor‒product relationship means that stress-induced upregulation of serotonin biosynthesis automatically expands the substrate pool available for melatonin production, establishing a potential indoleamine amplification loop where an initial serotonin signal can be metabolically extended and transformed into a melatonin-mediated response.^23^^,^^43^ However, their roles are far from redundant. Emerging evidence points to temporal and spatial specialization in their mode of action. Serotonin often functions as a first responder or a local signal, accumulating rapidly at the precise site of stress imposition—such as a wound site, a root encountering salt, or a leaf under UV exposure—to provide immediate redox control, initiate local changes in ion flux, and modulate hormone signaling in real time.^2^ Its actions are frequently rapid and confined. In contrast, melatonin, whose synthesis may follow with a slight delay as the serotonin pool is metabolized, appears to function more in longer-term, systemic adaptation. It is often involved in integrating stress signals over time and across different plant tissues, reinforcing circadian rhythms disrupted by stress, and coordinating sustained physiological adjustments such as prolonged antioxidant defense or systemic acquired resistance.^29^^,^^31^^,^^44^

Beyond temporal dynamics, serotonin and melatonin exhibit divergent physiological emphases and molecular targets, despite shared functions like enhancing antioxidant capacity. A defining and specific function of serotonin is its potent role as a modulator of auxin signaling, particularly acting as a natural auxin inhibitor in roots to repress primary growth and stimulate lateral rooting, a critical function for stress-induced architectural plasticity.^3^ This direct and strong interference with auxin transport and response is not a major reported function of melatonin. Conversely, melatonin has been more strongly and consistently linked to the regulation of specific mitogen-activated protein kinase (MAPK) cascades and components of the ubiquitin-proteasome system in stress contexts, pathways that are less emphasized in serotonin's action profile.^31^^,^^45^ Their interaction with hormone pathways also reveals distinct nuances; while both influence abscisic acid (ABA) and jasmonic acid (JA)/salicylic acid (SA) balances, serotonin's effect can be more sharply antagonistic or synergistic in a pathogen-specific manner, as seen in rice where it suppresses JA signaling to prioritize SA defenses against certain pathogens.^26^ Melatonin's modulation of these hormones often appears more modulatory and less sharply switching.

Despite these distinctions, their partnership is overwhelmingly synergistic and often agonistic. In numerous applied studies, the combined application of serotonin and melatonin yields additive or even synergistic improvements in stress tolerance that exceed the effect of either compound alone, robustly supporting the concept of a coordinated indoleamine stress-response module.^2^ This synergy likely arises from their sequential action and complementary specializations: serotonin's rapid, localized intervention stabilizes the immediate cellular environment, creating the conditions (and possibly the metabolic substrate) for melatonin to effectively orchestrate a more comprehensive, plant-wide adaptive response. Understanding the environmental and genetic factors that regulate the metabolic flux from serotonin to melatonin—such as light conditions, temperature, and the expression levels of SNAT and ASMT—is therefore key to manipulating this module for agricultural benefit. In summary, the serotonin–melatonin relationship does not one of overlap but of elegant complementarity. It is a partnership built on biochemical continuity, temporal succession, and functional specialization, where serotonin's role as an acute, localized signaler and melatonin's role as a chronic, systemic integrator combine to provide plants with a layered and highly effective defense strategy against a wide spectrum of environmental challenges.

## 7. Conclusion: serotonin as the architect of plant stress resilience

The comprehensive synthesis presented in this review unequivocally establishes serotonin as far more than a stress-associated metabolite; it is a central integrator and master architect of plant stress adaptation. Operating at the critical nexus of redox chemistry, hormone network interaction, ion transport regulation, and gene expression control, serotonin enables plants to execute sophisticated, context-dependent decisions that dynamically balance growth with defense. This review has moved decisively beyond a fragmented catalog of antioxidant correlations to present a unified mechanistic framework, illuminating serotonin's role as a hormonal signal modulator, a precursor within a broader indoleamine network, and a direct agent of biotic defense. By critically distinguishing its temporal and functional specialization from melatonin—positioning serotonin as the rapid, localized responder and melatonin as the sustained, systemic integrator—we provide a clarified, nuanced understanding of this essential signaling module. The translational potential of this knowledge is significant and multifaceted, spanning the development of serotonin-based biostimulants for priming crops, genetic engineering of its biosynthetic pathways, and innovative microbiome engineering strategies that leverage its role as a rhizosphere signal. However, the future trajectory of this field hinges on bridging profound fundamental gaps, most notably the elusive identity of serotonin receptors or high-affinity binding proteins in plants, which remains the paramount unanswered question essential for decoding initial perception. Further research must also elucidate the downstream signaling specificity that coordinates its diverse effects and rigorously evaluate the long-term ecological consequences of manipulating serotonin pathways in agricultural systems. Success in these endeavors will not only complete our foundational understanding of plant chemical ecology but will also unlock the full potential of serotonin-informed strategies. In an era defined by escalating climatic volatility and environmental degradation, harnessing the sophisticated regulatory power of this ancient molecule offers a promising, sustainable pathway to engineer crop resilience, safeguard global food security, and illuminate the remarkable adaptive intelligence of the plant kingdom.

## Data Availability

All data generated during this research are included in this published article. The analysis during the study can be obtained from the corresponding author Hamzeh Amiri on reasonable request.
